# Apoptosis in capillary endothelial cells in ageing skeletal muscle

**DOI:** 10.1111/acel.12169

**Published:** 2013-11-19

**Authors:** Huijuan Wang, Anne Listrat, Bruno Meunier, Marine Gueugneau, Cécile Coudy-Gandilhon, Lydie Combaret, Daniel Taillandier, Cécile Polge, Didier Attaix, Claire Lethias, Kijoon Lee, Kheng Lim Goh, Daniel Béchet

**Affiliations:** 1 INRA, UMR 1019, Unité de Nutrition Humaine, CRNH AuvergneF-63122, Saint Genès Champanelle, France; 2 School of Chemical and Biomedical Engineering, Nanyang Technological UniversitySingapore, Singapore; 3 INRA-Vetagro Sup, UMR 1213, Unité Mixte de Recherche sur les HerbivoresF-63122, Saint Genès Champanelle, France; 4 Clermont Université, Université d’AuvergneF-63000, Clermont-Ferrand, France; 5 UMR5305, Laboratoire de Biologie Tissulaire et Ingénierie, Institut de Biologie et Chimie des Protéines, CNRS-Université de LyonF-69367, Lyon, France; 6 School of Mechanical and Systems Engineering, Newcastle University InternationalSingapore, Singapore

**Keywords:** angiogenesis, apoptosis, sarcopenia, satellite stem cell

## Abstract

The age-related loss of skeletal muscle mass and function (sarcopenia) is a consistent hallmark of ageing. Apoptosis plays an important role in muscle atrophy, and the intent of this study was to specify whether apoptosis is restricted to myofibre nuclei (myonuclei) or occurs in satellite cells or stromal cells of extracellular matrix (ECM). Sarcopenia in mouse gastrocnemius muscle was characterized by myofibre atrophy, oxidative type grouping, delocalization of myonuclei and ECM fibrosis. Terminal deoxynucleotidyl transferase-mediated dUTP nick end-labelling (TUNEL) indicated a sharp rise in apoptosis during ageing. TUNEL coupled with immunostaining for dystrophin, paired box protein-7 (Pax7) or laminin-2α, respectively, was used to identify apoptosis in myonuclei, satellite cells and stromal cells. In adult muscle, apoptosis was not detected in myofibres, but was restricted to stromal cells. Moreover, the age-related rise in apoptotic nuclei was essentially due to stromal cells. Myofibre-associated apoptosis nevertheless occurred in old muscle, but represented < 20% of the total muscle apoptosis. Specifically, apoptosis in old muscle affected a small proportion (0.8%) of the myonuclei, but a large part (46%) of the Pax7^+^ satellite cells. TUNEL coupled with CD31 immunostaining further attributed stromal apoptosis to capillary endothelial cells. Age-dependent rise in apoptotic capillary endothelial cells was concomitant with altered levels of key angiogenic regulators, perlecan and a perlecan domain V (endorepellin) proteolytic product. Collectively, our results indicate that sarcopenia is associated with apoptosis of satellite cells and impairment of capillary functions, which is likely to contribute to the decline in muscle mass and functionality during ageing.

## Introduction

Sarcopenia is the progressive generalized loss of skeletal muscle mass and function, which occurs as a consequence of ageing. Sarcopenia results in impaired locomotion and is associated with an increased susceptibility to illness, as muscle is a major site of fatty acid oxidation and carbohydrate metabolism and a body reservoir of readily available amino acids. Multiple phenomena are involved in the development of sarcopenia. In humans, intrinsic factors include altered hormonal levels (menopause, andropause, adrenopause, somatopause), increased levels of inflammatory cytokines (Lamberts *et al*., 1997), neuronal remodelling (Lexell, 1997), fibrosis of the extracellular matrix (ECM) (Kragstrup *et al*., 2011), and impaired microvascular (Herrera *et al*., 2010) and satellite cell functions (Renault *et al*., 2002). Extrinsic factors such as a poor nutritional status or physical activity also play major roles in the aetiology of sarcopenia (Valdez *et al*., 2010).

From a histological perspective, muscle ageing is characterized by a decrease in myofibre size and number, with a preferential loss of type II myofibres. At the myofibrillar level, important modifications in contractile and cytoskeletal components, and in essential regulatory proteins, likely account for dysfunctions in old muscle contraction, as shown in rodents (Piec *et al*., 2005). Other features support perturbations in protein turnover (Combaret *et al*., 2009), reduced energy metabolism and altered detoxification of reactive oxygen species (Baraibar *et al*., 2013). Obviously, the mechanisms relating to sarcopenia are complex and probably result from the alteration of a variety of interrelated cellular functions (Ibebunjo *et al*., 2013).

Although more information is available about the mechanisms that affect contractile myofibres during ageing, few studies have investigated the implication of ECM embedding myofibres. ECM is a critical component in the transfer of force from the muscle myofibre out to the tendon and subsequent bone (Voermans *et al*., 2008). ECM plays an important role in maintaining the structure of the muscle and also in providing an environment in which the contractile myofibres can function. ECM also contains different types of stromal cells such as fibroblasts, immune cells, adipocytes and capillary cells, which reciprocally are involved in the turnover of ECM and in the regulation of myofibre metabolism (Kragstrup *et al*., 2011).

Recent evidence indicates that apoptosis is involved in mediating the progression of sarcopenia (Marzetti *et al*., 2010). There is also increasing knowledge about the pathways and effectors of the apoptotic process in skeletal muscle. However, it is not fully understood whether age-dependent apoptosis is mainly restricted to myofibre nuclei (myonuclei) or whether apoptosis also occurs in other muscle cells. Herein, we report that apoptosis in old skeletal muscle is not confined to myofibres, but is preponderant in capillary endothelial cells and of significant importance in satellite cells. We further provide evidence that ECM remodelling is characterized by a limited proteolysis of the basement membrane perlecan and with the production of endorepellin fragments.

## Results

### Age-dependent modifications in myofibre morphology

**Figure 1:**
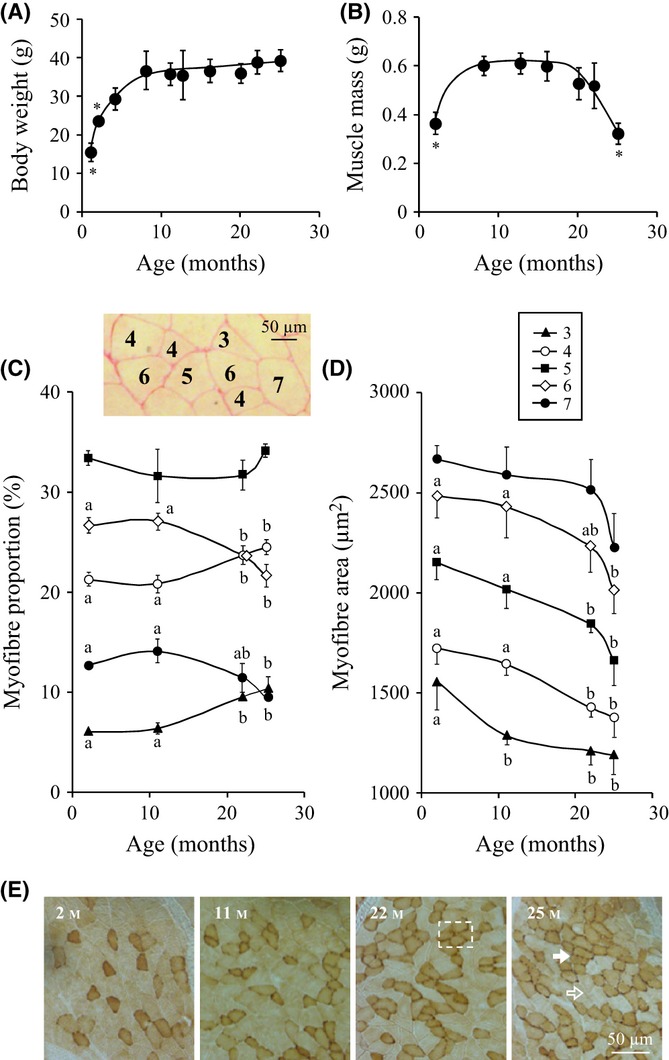
Sarcopenia in C57BL6 mice and myofibre morphology. (A) Body weight and (B) *gastrocnemius* muscle (GM) mass for 2–25 months old C57BL6 mice. Muscle cross sections were stained with Sirius red, and 800 individual myofibres were analysed per mouse and classified according to the number of neighbour myofibres. Age-dependent changes in the (C) distribution and (D) cross-sectional area of myofibres with 3, 4, 5, 6 or 7 neighbours in 2–25 months GM. All values in the graphs are means ± SEM (vertical bars) for *n* = 4 mice per age, and different letters indicate significant difference (*P* < 0.05) between ages. (E) Images of GM cross sections stained for cytochrome oxidase (Cox) in 2–25 months mice. Cox-positive (solid arrow) and Cox-negative (hollow arrow) myofibres are indicated. Region of interest (dotted box) in 22 months muscle cross section highlights oxidative myofibre grouping.

The preferential loss of *gastrocnemius* muscle (GM) muscle mass is shown in Fig. 1. Body weight increased during maturation, between 2 and 11 months, and then remained stable until 25 months (Fig. 1A). GM mass similarly raised during maturation was maintained in adult mice, but strongly decreased during ageing (from 20 to 25 months) (Fig. 1B). This marked atrophy of GM muscle reflected sarcopenia in old mice.

Age-dependent muscle atrophy was associated with modifications in myofibre morphology, so that old myofibres appeared less polygonal, that is, surrounded by less neighbouring myofibres. To quantify this observation, we classified myofibres according to their number of neighbours and measured cross-sectional areas for about 800 individual myofibres per mouse. In the young adult (2 months), mature adult (11 months), early old (22 months) and advanced old (25 months) GM, myofibres with five neighbours were always the most abundant. However, during ageing, the proportion of myofibres with six and seven neighbours decreased, while symmetrically the myofibres with three and four neighbours increased, suggesting that myofibres became more acute in shape (Fig. 1C). As expected, larger myofibres had more neighbours, and as shown in Fig. 1(D), the most abundant classes of myofibres (four to six neighbours) exhibited an age-related decrease in cross-sectional area.

Cytochrome c oxidase (Cox), a marker for oxidative energy metabolism, characterizes slow contracting myofibres. In mice GM, Cox preferentially labelled the small myofibres with three to five neighbours. As shown in Fig. 1(E), Cox myofibres presented a chessboard-like distribution in muscle cross sections of 2 and 11 months adult muscles, while myofibre-type grouping was clearly apparent in 22 and 25 months old muscles. Therefore, GM ageing in mice was associated with atrophy and grouping of acute-shaped myofibres.

### Age-dependent modifications in the various populations of muscle nuclei

Skeletal muscles contain different cellular populations: multinucleated myofibres, satellite cells and stromal cells of ECM. Stromal cells are located outside the basal lamina, while most satellite cells are located between the myofibre sarcolemma and basal lamina (Scharner & Zammit, 2011). Further studies were then performed to specify which cellular population is mainly affected by ageing.

**Figure 2:**
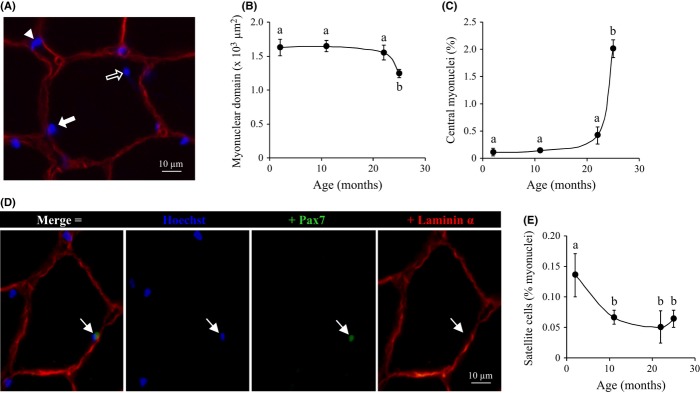
Myofibre nuclei during ageing of mouse muscle. (A) Representative image of a 25 months muscle cross section labelled for nuclei (Hoechst 33258, blue) and plasma membrane dystrophin (red). A peripheral myonucleus (solid arrow), internal myonucleus (hallow arrow) and stromal cell nucleus (arrow head) are indicated. Age-related variations in (B) myonuclear domain (myofibre area controlled per myonucleus), and (C) proportion of internal myonuclei. (D) Immunolocalization of a satellite cell in a 2 months muscle cross section colabelled with Hoechst 3258 (blue), anti-paired box protein-7 (Pax7) (satellite cells, green) and anti-laminin 2α (basal lamina, red). (E) Proportion of satellite cell nuclei among total myofibre nuclei. Values in the graphs are means ± SEM (*n* = 4), and different letters indicate significant difference (*P* < 0.05) between ages.

Hoechst staining of nuclei and colabelling of the sarcolemma with anti-dystrophin were used to distinguish nuclei in myofibres (myonuclei; Fig. 2A). These analyses indicated that myofibres maintained a similar content of myonuclei with age (*P* > 0.42). However, because of smaller cross-sectional area, the myonuclear domain (the myofibre area controlled per myonucleus) significantly decreased in 25 months muscles (Fig. 2B; *P* < 0.03). While myonuclei are typically located beneath the sarcolemma in young and adult myofibres, a characteristic feature of the old muscle was also a sharp rise in centrally located myonuclei (Fig. 2C; *P* < 0.001). Hoechst staining of total muscle nuclei and costaining of the basal lamina further indicated that, outside the basal lamina, stromal cell nuclei (Fig. 2A, arrow head) represented a significant proportion of total muscle nuclei. However, this proportion of stromal nuclei remained unchanged during ageing (48.6 ± 3.2 vs. 44.7 ± 2.8% in 11 and 25 months muscles, respectively, *P* = 0.25).

The nuclear transcription factor paired box protein-7 (Pax7) is a reliable marker of both quiescent and activated satellite cells (Péault *et al*., 2007), and anti-Pax7 was then used to investigate these muscle stem cells. Triple labelling with Hoechst, anti-Pax7 and anti-laminin 2α confirmed that Pax7^+^ satellite cell nuclei were located beneath the basal lamina (Fig. 2D). Our study of Pax7^+^ cells in GM cross sections from young adult to old mice indicated that the proportion of satellite cells strongly decreased (−51%; *P* < 0.05) during maturation (2–11 months), but remained at a low level thereafter in adult and old muscles (Fig. 2E).

### Apoptosis essentially occurs in extracellular matrix stromal cells

**Figure 3:**
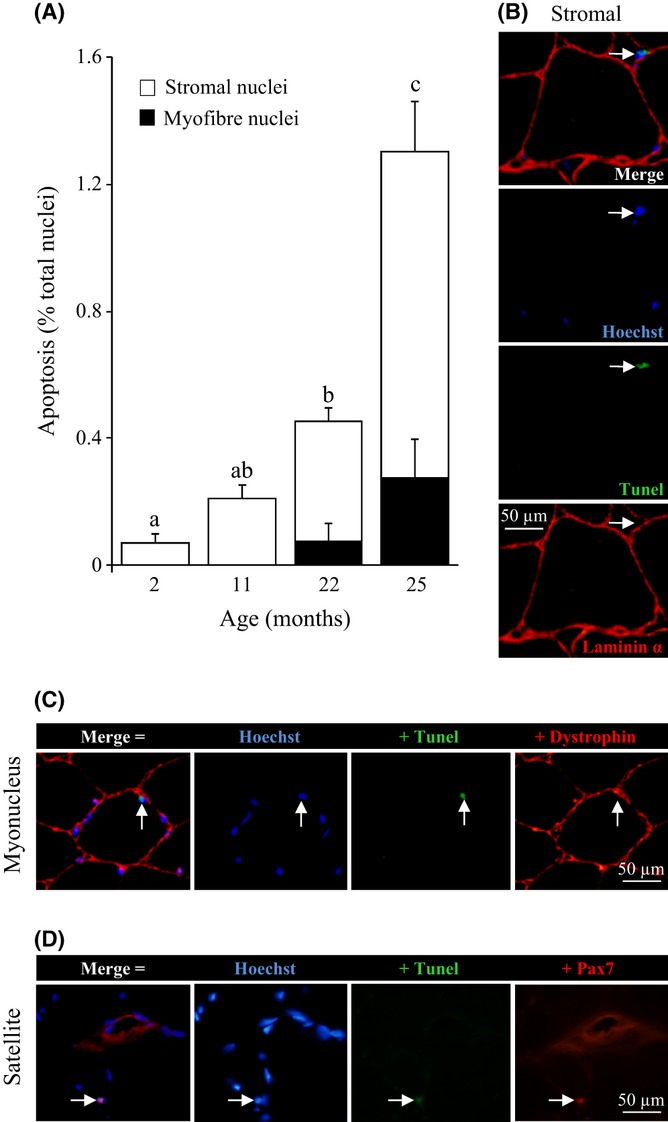
Increased apoptosis in old mouse muscle. (A) Proportion of apoptotic nuclei in 2–25 months mice *gastrocnemius* muscle as revealed by terminal deoxynucleotidyl transferase-mediated dUTP nick end-labelling (TUNEL). Colabelling for laminin 2α distinguishes apoptosis in myofibers (myonucleus and satellite, black bars) and stromal cells (white bars). Values are means ± SEM (*n* = 4), and different letters indicate significant difference (*P* < 0.05) between ages. (B) Representative images of a 25 months muscle cross section labelled with Hoechst 33258 (blue), TUNEL (green) and anti-laminin 2α (red); the arrow indicates an apoptotic stromal cell nucleus. (C) Apoptotic myonucleus (arrow) in 25 months muscle cross section colabelled for Hoechst 33258 (nuclei, blue), apoptosis (TUNEL, green) and dystrophin (plasma membrane, red). (D) Apoptotic satellite cell (arrow) in 25 months muscle cross section co-labelled for Hoechst 33258 (nuclei, blue), apoptosis (TUNEL, green) and paired box protein-7 (Pax7) (satellite cell, red).

Because the modifications that occur in muscle during ageing might be related to apoptosis, we performed terminal deoxynucleotidyl transferase-mediated dUTP nick end-labelling (TUNEL) analyses. As shown in Fig. 3(A), TUNEL^+^ nuclei strongly increased in the old muscle (*P* < 0.001). To further distinguish whether apoptosis occurred in myofibres or in ECM stromal cells, immunostaining of laminin 2α was performed together with TUNEL and Hoechst (Fig. 3B). Interestingly, apoptotic nuclei in adult mice essentially belonged to stromal cells in the connective tissue. Moreover, the age-dependent rise in apoptotic nuclei was mostly attributed to stromal cells (Fig. 3A, white bars). TUNEL studies coupled with Hoechst and laminin 2α immunostaining nonetheless indicated that apoptosis also occurred in myofibre-associated nuclei (myonuclei and/or satellite nuclei), but only in old muscles (< 20% of total muscle apoptosis; Fig. 3A, black bars).

### Apoptosis in myofibres and in satellite cells

Further studies were then performed with 25 months muscles to specify whether myofibre-associated apoptosis was due to myonuclei and/or to satellite cells. Muscle cross sections were immunolabelled for dystrophin to specifically localize the sarcolemma and to distinguish myonuclei (inside the sarcolemma), from satellite and stromal nuclei (outside the sarcolemma) (Fig. 3C). Triple labelling with TUNEL, Hoechst and anti-dystrophin of GM cross sections was carried out for four old mice. More than 1200 myofibres were assessed per mouse, but apoptosis was found to affect only a limited proportion (0.78 ± 0.15%, *n* = 4) of the total myofibre myonuclei.

To identify apoptotic satellite cells, cross sections of 25 months muscle were triple labelled with TUNEL, Hoechst and anti-Pax7 (Fig. 3D). Of the 17 satellite cell nuclei identified in the old GM of four mice, eight were found to be apoptotic. Therefore, our study revealed the existence of TUNEL^+^ satellite cells and strikingly that apoptosis occurred for a significant proportion (45.8 ± 6.7%, *n* = 4) of the Pax7^+^ satellite cells in GM from 25 months old mice.

### Apoptosis in capillary endothelial cells

**Figure 4:**
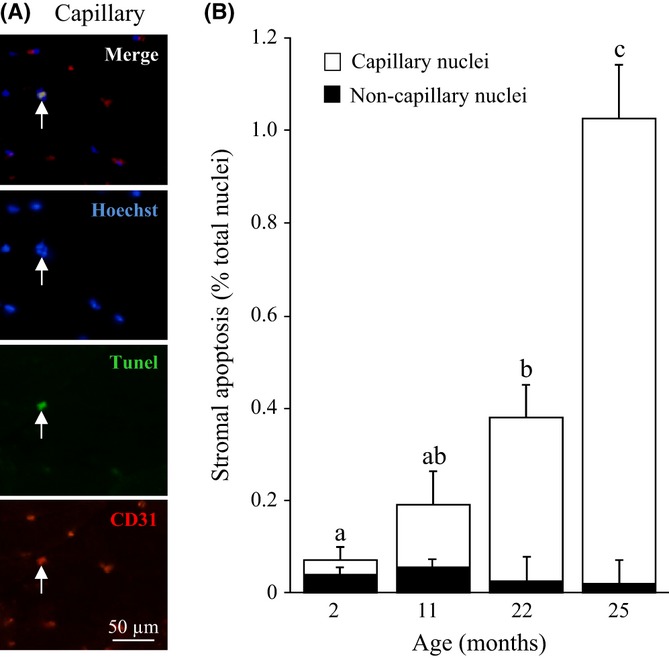
Apoptosis in capillary endothelial cells. (A) Apoptotic capillary endothelial cell (arrow) in 25 months muscle cross section co-labelled for Hoechst 33258 (nuclei, blue), apoptosis (TUNEL, green) and CD31 (capillary endothelial cell, red). (B) Age-dependent increase in stromal and capillary endothelial cell apoptosis (white bars). Few non-capillary stromal apoptotic cells (black bars) remain to be identified. Values are means ± SEM (*n* = 4), and different letters indicate significant difference (*P* < 0.05) between ages.

Because TUNEL-positive nuclei were predominantly found in stromal cells in adult and old GM, additional investigations were carried out to identify which cellular population is apoptotic in ECM. ECM contains different categories of stromal cells, including fibroblasts, adipocytes, capillary cells and immune cells. Macrophages or other white blood cells could rarely be observed in our muscle cross sections. CD31 is highly expressed on endothelial cells and is a major constituent of the intercellular junction in confluent vascular beds (Privratsky *et al*., 2010). Anti-CD31 is therefore commonly used to mark capillary endothelial cells (Christov *et al*., 2007). Triple labelling of muscle cross sections with TUNEL, Hoechst and CD31 was then used to label apoptotic nuclei, total nuclei and capillary endothelial cells, respectively (Fig. 4A). These studies indicated that in adult GM, a significant proportion of the apoptotic stromal cells were CD31^+^ capillary endothelial cells (Fig. 4B). Moreover, our data revealed that the age-dependent rise in stromal cell death was mostly due to the apoptosis of capillary endothelial cells (Fig. 4B). CD31 labelling of capillaries also showed that, despite increased endothelial cell apoptosis, the ratio of capillary density to myofibre density was not significantly (*P* > 0.6) altered by ageing.

### Age-dependent modifications in extracellular matrix structure

**Figure 5:**
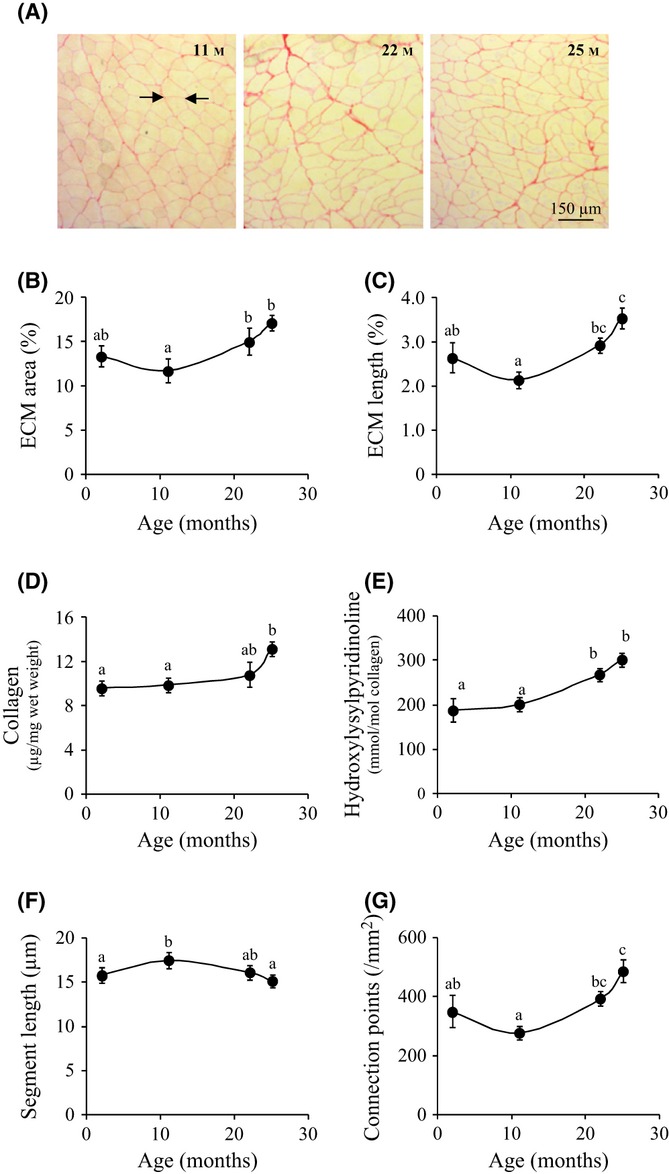
Age-dependent variations in extracellular matrix (ECM) characteristics. (A) Images of *gastrocnemius* muscle cross sections stained with Sirius red at 11, 22 and 25 months. Myofibres and ECM are yellow and red, respectively. A connection point (arrow) is defined as the interaction between ≥ 3 myofibres, and each ECM segment is located between two connection points. Age-related variations in (B) ECM total area (% of total area), (C) ECM total length (% of total area), (D) collagen content, (E) hydroxylysylpyridinoline content, (F) ECM mean segment length and (G) number of connection points. All values in the graphs are means ± SEM (vertical bars) for *n* = 4 mice per age, and different letters indicate significant difference (*P* < 0.05) between ages.

Alterations in myofibre size and morphology, together with apoptosis in stromal cells, were in agreement with the profound remodelling of ECM that we observed during ageing (Fig. 5A). To characterize ECM modifications, image analyses were performed with muscle cross sections stained with Sirius red which labels major ECM components, type I and type III collagens (Tullberg-Reinert & Jundt, 1999). The total area (Fig. 5B) and total length (Fig. 5C) of ECM skeleton in GM cross sections both showed similar U-shaped evolutions (*P* < 0.02) with a minimum value at 11 months, and a maximum at 25 months. The muscle content of collagen hydroxyproline also increased in 25 months muscle (Fig. 5D, *P* < 0.02), which confirmed the emergence of ECM fibrosis in the old muscle. Additionally, old GM revealed enrichment in non-reducible hydroxylysylpyridinoline collagen cross-linking (Fig. 5E, *P* < 0.01) implicating an increased muscle stiffness.

The atrophy and reduced cross-sectional area of the old myofibres might be expected to be associated with an increased ramification of ECM embedding myofibres. Therefore, we further analysed ECM segments between pair of myofibres. While the mean length of ECM segments decreased (Fig. 5F, *P* < 0.03), the density of ECM connection points (where ≥ 3 segments interact) increased during ageing (Fig. 5G, *P* < 0.02), which confirmed an increased ramification of ECM.

### Age-dependent modifications in extracellular matrix components

**Figure 6:**
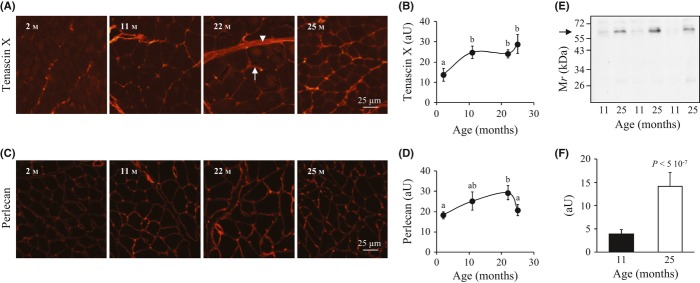
Age-related variations in tenascin-X and perlecan. Representative images of *gastrocnemius* muscle cross sections from 2- to 25-month-old mice immunolabelled with (A) anti-tenascin-X and (C) anti-perlecan; perimysium (arrow head) and endomysium (solid arrow) are indicated. Semi-quantitative estimation of (B) tenascin-X and (D) perlecan immunolabelling in muscles during ageing. (E) Whole muscle lysates from 11 and 25 months muscles were analysed by Western blotting using a monoclonal anti-endorepellin antibody; the arrow indicates the LG1-LG2 endorepellin fragment; (F) corresponding densitometry analysis of the blot showing the increase in LG1-LG2 peptide.

To further assess changes in ECM at the molecular level, several ECM components were investigated. Type VI collagen and tenascin-X are known to be localized both in the endomysium and the perimysium, while type IV collagen, laminin 2α and perlecan are exclusively located in the endomysial basement membrane (Voermans *et al*., 2008). Semi-quantitative immunohistochemistry of type IV and VI collagens and of laminin 2α did not reveal significant changes during ageing (data not shown). However, age-dependent modifications were observed for tenascin-X and perlecan immunolabelling in the endomysium, but with distinct timings. Tenascin-X, which determines the mechanical properties of collagen (Margaron *et al*., 2010), increased between 2 and 11 months, and remained stable thereafter (Fig. 6A,B, *P* < 0.03). In contrast, perlecan immunolabelling progressively reached a maximum at 22 months and decreased at 25 months (Fig. 6C,D, *P* < 0.05).

Previous studies on capillaries reported that apoptosis of endothelial cells is associated with a limited proteolysis of perlecan domain V (endorepellin) and with the production of peptides (called LG1–LG2 and LG3) that might be important for fibrosis of ECM (Laplante *et al*., 2005). Because we observed that ageing in mice skeletal muscle was associated with (i) enhanced apoptosis of capillary endothelial cells, (ii) altered expression of perlecan and (iii) increased ECM fibrosis, we hypothesized that endorepellin fragments might be produced in old muscles. Western blots of total muscle extracts with a specific antibody to endorepellin identified a 63-kDa fragment (Fig. 6E) that corresponds to endorepellin LG1–LG2 and revealed that GM ageing was associated with a sharp increase in this endorepellin 63 kDa fragment (Fig. 6F).

## Discussion

Age-dependent atrophy of skeletal muscle is associated with profound alterations in myofibres and with remodelling of the connective tissue embedding myofibres. The diminution in myofibre cross-sectional area and nuclear domain, together with myofibre-type grouping that we observed in the GM of old mice, are in agreement with the previous studies on human (Andersen, 2003) and rat (Yarovaya *et al*., 2002) skeletal muscles. Image analyses were further used in the present study to demonstrate enhanced angularity, a notion previously mentioned but not specifically quantified in old muscle (Andersen, 2003).

The increased stiffness and reduced function of the old muscle are also associated with ECM fibrosis and increased collagen concentration and cross-linking (Kragstrup *et al*., 2011). Herein we further extend this notion using image analyses to provide evidence that fibrosis is associated with thickening of ECM endomysium and an increased ramification of the ECM, which is required for myofibre atrophy.

### Myonuclear apoptosis is a rare event in the old muscle

Age-dependent alterations in myofibre and ECM morphology were associated with modifications in the different populations of nuclei belonging to myofibres and to muscle connective tissue. Myofibre myonuclei presented a reduced myonuclear domain and an increased centralization in old skeletal muscle. Centralized myonuclei are recognized markers of regenerating myofibres (Yablonka-Reuveni & Anderson, 2006). However, in the old atrophying muscle, centralized myonuclei could also result from myofibre denervation and myofibre branching (Valdez *et al*., 2010). In addition, alterations in the microtubule network during ageing (Piec *et al*., 2005) might also cause changes in the distribution of the myonuclei (Bruusgaard *et al*., 2006). Previous investigations about cell death in old skeletal muscle suggested that apoptotic nuclei were mostly myonuclei, although the identification of the nuclei has not been unambiguous. Our current study in mice indicates that myonuclear apoptosis could not be detected and thus is a rare event in young and adult GM. Nonetheless, apoptotic myonuclei did appear during ageing, although these did not account for more than 20% of total apoptosis in the old mouse GM. Myonuclear apoptosis is therefore a rare event also in old mouse muscle (0.8% of total myonuclei), which may nonetheless be important for the functionality of myofibres (Marzetti *et al*., 2010).

### Increased apoptosis of satellite cells in the old muscle

Age-related muscle loss is believed to partly result from the diminishing ability of muscle to repair itself (Shavlakadze *et al*., 2010). Satellite cells function as myogenic progenitors in adult muscles, and an age-linked decline in satellite cell regenerative potential may limit repair of old muscles. The capacity of satellite cells to support muscle maintenance depends on their abundance, on their myogenic potential and on their local environment. In GM, the major decrease in satellite cell abundance occurs by 11 months of age during maturation, that is, before ageing. Previous studies performed in C57BL6 mice similarly showed that in extensor digitorum longus muscle, satellite cell number decreases before 1 year of age (Shefer *et al*., 2006). While satellite cell number remained at a low level in old muscles, we also provide evidence that apoptosis occurs for a significant proportion of old satellite cells. This is in agreement with indications that satellite cells derived from old rat muscle demonstrate an increased susceptibility to apoptosis *in vitro* (Jejurikar *et al*., 2006). Previous studies have demonstrated increased apoptotic satellite cells in response to exercise (Podhorska-Okolow *et al*., 1998) and denervation (Bruusgaard & Gundersen, 2008) in limb muscles, but the importance of ageing for satellite cell apoptosis was only acknowledged in thyroarytenoid muscle for laryngeal function (Malmgren *et al*., 2001). Our observations in GM now indicate that satellite cell apoptosis is also associated with age-related loss of limb muscles. Such apoptosis likely contributes to the decline of satellite cells with age, but may not result in major functional deficit. Previous studies indeed emphasized that the few satellite cells that survive the effects of ageing retain a full potential for muscle regeneration (Collins *et al*., 2007; Shavlakadze *et al*., 2010; Alsharidah *et al*., 2013). In old mice muscle, the reduced number of Pax^+^ satellite cells is partly counterbalanced by non-myogenic cells located in the same niche, but the function and origin of those cells is not clarified (Collins *et al*., 2007).

### Capillary endothelial cells contribute to apoptosis in the adult muscle

The present study further emphasizes that besides myofibre myonuclei and satellite cells, stromal cells of ECM account for a significant part of total nuclei in mice GM. Similar estimations of stromal cell proportion were provided for rat (Schmalbruch & Hellhammer, 1977) and mice (Murray & Robbins, 1982) skeletal muscles. Although stromal cells comprise 30–40% of the total nuclei in adult rodent skeletal muscle, their contribution to apoptosis nevertheless has been largely neglected. Apoptotic nuclei are rare in normal adult mice GM, and in accordance with the previous studies in adult rat muscle (Allen *et al*., 1997), our triple labelling (lamina, TUNEL, Hoechst) showed that the majority of apoptotic nuclei were located in ECM in the adult muscle. Our study now identifies these rare apoptotic cells in adult muscle as CD31^+^ capillary endothelial cells.

### Capillary endothelial cells contribute to apoptosis in the old muscle

Previous studies in different models of atrophying muscles reported that apoptosis is not confined to myofibres, as apoptosis occurs in stromal cells in response to underweighting (Allen *et al*., 1997), hypertension (Gobé *et al*., 1997), heart failure (Vescovo *et al*., 1998) and in dystrophic muscles (Sandri *et al*., 1998). However, the implication of stromal cells in muscle apoptosis was not previously mentioned during the ageing process. While ageing was associated with a detectable but limited rise of apoptosis in myonuclei, we provide evidences that apoptotic cells in old GM are mostly located in ECM. Moreover, our data identify CD31^+^ capillary endothelial cells as the major apoptotic cells in sarcopenic muscle. Apoptosis was observed in muscle capillaries in response to high salt intake in rats (de Resende *et al*., 2006), but was not previously reported in capillaries of aged skeletal muscle.

Ageing predisposes to a progressive impairment of the vasculature, and several studies reported the deleterious impact of age on endothelial functions in the peripheral microcirculation (Herrera *et al*., 2010; Virdis *et al*., 2010). One mechanism for this endothelial dysfunction is the increased density of apoptotic endothelial cells (Asai *et al*., 2000). During vascular remodelling, apoptosis in endothelial cells triggers the release of fragments of endorepellin (perlecan domain V) (Laplante *et al*., 2005). Endorepellin contains three laminin-like globular domains (LG1 to LG3). While LG3 relatively freely diffuses and can be detected in many body fluids, LG1–LG2 binds specifically to major basement membrane constituents (Whitelock *et al*., 2008; Iozzo *et al*., 2009). In agreement with these observations, we report that apoptosis of capillary endothelial cells in the old skeletal muscle is coincident with the production of LG1–LG2 endorepellin fragment. However, we cannot specify whether muscle cells, other than endothelial cells, also contribute to the production of LG1–LG2 fragment. Matrix proteases, such as plasmin, collagenase and stromelysin (Whitelock *et al*., 2008), but also oxidative damage (Rees *et al*., 2010), could be involved in partial proteolysis of perlecan, while bone morphogenetic protein-1 (BMP-1/Tolloid) or cathepsin L could cleave LG3 from endorepellin/perlecan (Whitelock *et al*., 2008).

Our study indicates that apoptosis of capillary endothelial cells accounts for more than 75% of apoptosis in the old mouse muscle. Regeneration mechanisms, such as division or hyperplasia of adjacent endothelial cells, or joint of circulating endothelial progenitor cells (Herrera *et al*., 2010), are also likely involved to maintain capillary to myofibre ratios in the old muscle. Overall, these phenomena may be of importance given the fundamental role of endothelial cells in the regulation of vascular homeostasis. Of note, blood vessels and satellite cells could be in close vicinity, and satellite cells may influence angiogenesis, and reciprocally, endothelial cells may enhance satellite cell growth (Christov *et al*., 2007). Strikingly, these two cell populations were the most affected by apoptosis in sarcopenic muscle of old mice. If circulating factors trigger cell damage, capillary endothelial cells and juxtavascular satellite cells may be subjected to harmful stimuli to a higher degree than myofibres. In turn, dysfunction in endothelial cell turnover could disturb the integrity of the endothelial monolayer and produce profound alterations in the delivery of nutrients and oxygen and in the removal of toxic metabolic products.

## Experimental procedures

### Animals, muscle samples

C57BL6 male mice (Jackson Laboratory, Singapore, Singapore) were from the Laboratory Animal Centre of the National University of Singapore and raised in the veterinarian-staffed Laboratory Animal Facility at Nanyang Technological University (NTU) following the procedure of the Institutional Animal Care-and-Use Committee. Mice were housed in a temperature (22 ± 1 °C)- and humidity (50–70%)-controlled facility, with a 12:12 h light–dark cycle, and food (SAFE, Singapore, Singapore) and water were provided *ad libitum*. Mice were either 2, 11, 22 or 25 months of age, corresponding to young adult, mature adult, early old or advanced old mice, respectively. Mice were killed by cervical dislocation after CO_2_ anaesthesia and weighted, and GM were rapidly removed from both hind limbs and weighted. Samples from the mid-belly of the lateral and medial heads of GM were used. Muscle samples were either snap-frozen in liquid nitrogen or for histological analyses mounted on cork board and frozen in isopentane cooled on liquid nitrogen. Four serial cross sections (10 μm thickness) were collected at 100-μm intervals throughout the entire sample using a cryostat (Microm, Francheville, France) at −25 °C.

### Muscle extracellular matrix and myofibres structure

Muscle ECM and myofibre characteristics were studied on cross sections stained with Sirius red (Tullberg-Reinert & Jundt, 1999). At least four cross sections (each corresponding to 100–250 myofibres) were analysed per mouse and for four to five mice per age. Sirius red stains ECM red and myofibres yellow, and provides important contrast between myofibres and ECM, well suitable for image analysis.

Images were captured with a DP-72 camera coupled to a BX-51 microscope (Olympus, Rungis, France) at a resolution of 0.32 or 0.16 μm per pixel. Five colour images per mouse were acquired under identical conditions (exposure time, white balance) in bright fields. Sirius red images were processed through a homemade visual basic program developed under visilog 6.7 software (Noesis, Gif sur Yvette, France). First, inverted optical density greyscale images were obtained from the green component of the colour images. Second, thresholding was carried out followed by skeletonization. Therefore, in the resulting binary image, ECM area and length, ECM segment length and number of connection points were derived out. A connection point was defined as the interaction between ≥ 3 myofibres and a segment as ECM link between two connection points. Reconstruction of binarized images was carried out and the total number of myofibres and characteristics of each myofibre (number of neighbours, cross-sectional area) were recorded.

Oxidative metabolism of myofibre was assayed by cytochrome c oxidase (Cox) histochemistry (Bio-Optica, Milan, Italy). Slow-twitch oxidative myofibres were Cox positive (dark brown), while fast-twitch glycolytic myofibres were Cox negative.

### Myonuclei

Total muscle nuclei were visualized on 10 μm cross sections stained with Hoechst 33258 (Sigma, L’Isle d’Abeau Chesnes, France). To distinguish myofibre nuclei (myonuclei), plasma membrane was labelled for dystrophin. After fixation with 4% paraformaldehyde for 10 min and antigenic site saturation (5% BSA in PBS) for 30 min, sections were incubated with primary rabbit anti-dystrophin (1:500; Abcam, Paris, France) for 1 h, rinsed with PBS three times and incubated with the secondary antibody conjugated to DyLight 488 (1:400; Interchim, Montluçon, France) for 45 min in the dark. Muscle sections were then washed twice with PBS, incubated 1 min with PBS containing 2 μg mL^−1^ Hoechst 33258 and mounted with Gel Mount (Sigma).

For all immunofluorescence analyses, images were captured with a DP-72 camera coupled to a BX-51 microscope at a resolution of 0.32 or 0.16 μm per pixel. Ten images per mouse, for four to five mice per age, were captured under optimal condition (exposure time) through adequate filters: blue (excitation at 345 nm, emission at 450–490 nm) for Hoechst 33258, green (excitation at 470 nm, emission at 510–560 nm) for DyLight 488 and red (excitation at 530 nm, emission at 575–650 nm) for DyLight 549. Negative controls were performed by omitting either the primary or secondary antibody on serial sections.

### Satellite cell nuclei

Satellite cell nuclei were identified by costaining with anti-Pax7 (paired box protein 7) and Hoechst 33258. Anti-Pax7 labels both quiescent and activated satellite cells (Péault *et al*., 2007). After saturation (5% BSA in PBS for 30 min), cross sections were incubated successively with 10% unconjugated AffiniPure Fab fragment anti-mouse IgG (H+L) (Interchim) for 1 h, with mouse monoclonal anti-Pax7 (1:50; Hybridoma Bank, Iowa City, IA, USA) for 1 h and with secondary antibody conjugated to DyLight 549 (1:400; Interchim) for 45 min. Hoechst 33258 staining was used to verify that Pax7 labelling corresponds to nuclei.

### Stromal cell nuclei

To distinguish ECM stromal nuclei, myofibre basal lamina was labelled for laminin 2α. After fixation with cold acetone for 10 min and antigenic site saturation (5% BSA in PBS) for 30 min, sections were incubated with primary rat anti-laminin 2α (1:200; Abcam) for 1 h, rinsed with PBS three times and incubated with the secondary antibody conjugated to DyLight 549 (1:400) for 45 min in the dark. Muscle sections were then washed twice with PBS, incubated 1 min with PBS containing 2 μg mL^−1^ Hoechst 33258 and mounted with Gel Mount.

### Apoptotic nuclei

Detection of apoptotic nuclei was performed using a TUNEL (Terminal deoxynucleotidyl transferase fluorescein-dUTP nick end-labelling) fluorescent detection kit (Roche Diagnostics, Meylan, France) according to the manufacturer’s instructions. TUNEL assay might also detect naturally occurring single-stranded DNA breaks, although this has mostly been reported in differentiating muscle cells (Larsen *et al*., 2010). This phenomenon is thus unlikely to be predominant in the old atrophying muscle, as myofibres not subjected to hypertrophic stimulus are refractory to satellite cell fusion. Apoptotic nuclei were counted and localized for approximately 1300 myofibres per mouse and for four mice per age. For all groups, positive controls were carried out on serial sections after prior incubation with 0.12 μg μL^−1^ DNAse I, while negative controls used labelling solution instead of TUNEL solutions. Costaining of apoptotic nuclei with anti-laminin 2α (1:200), anti-dystrophin (1:500) or anti-CD31 (1:100; Abcam) was performed after the TUNEL assay. Briefly, sections were rinsed with PBS for 30 min, saturated by 5% BSA in PBS for another 30 min, incubated with the primary antibody for 1 h and then with the secondary antibody conjugated to DyLight 549 for 45 min. Hoechst 33258 was also used to avoid false-positive TUNEL. Similar protocols were applied for costaining of apoptotic nuclei with Pax7, except that cross sections were pre-incubated overnight at 4 °C with unconjugated AffiniPure Fab fragment (1:20) before the incubation with mouse primary antibody.

### Extracellular matrix composition

Expression of several ECM components was studied by double indirect immunostaining on 10 μm thick sections for four animals per age. Guinea pig polyclonal anti-tenascin-X (1:100) was previously described (Margaron *et al*., 2010). Rabbit polyclonal antibodies to type IV collagen and type VI collagen (both 1:40; Novotec, Lyon, France), rat monoclonal to perlecan (1:1; Abcam), and guinea pig anti-tenascin-X were applied after saturating (5% BSA in PBS). Rat anti-laminin 2α was applied after acetone fixation and saturating. Incubations with corresponding DyLight 488- or 549-conjugated secondary antibodies (1:400) were for 45 min at room temperature**.** For each image, pixel intensity was randomly collected for 10 endomysial regions. Images were processed with ImageJ version 1.42q (NIH, Bethesda, MD, USA).

### Collagen and cross-link content

To estimate total collagen, frozen muscle powder (100 mg) was hydrolysed in 2 mL HCl 6 N overnight at 105 °C, incubated with activated charcoal (Norit A; Sigma) and diluted with four vol H_2_O. Hydroxyproline content was determined according to the procedure of Woessner (1961), and optical density was measured at 557 nm. Collagen content was calculated assuming that 14% amino acid in collagen is hydroxyproline.

Hydroxylysylpyridinoline cross-links were measured on the same hydrolysate. After 5 min centrifugation at 16 000 *g* and 4 °C, 300 μL of supernatants was added to 300 μL NaOH 6 N and 300 μL Tris 1 m. Final pH was adjusted between 6.5 and 7.5. Cross-links were determined in duplicate using the enzyme-linked immunoassay Metra Pyd EIA kit (Teco Medical, Paris, France) according to the manufacturer’s instructions. Cross-link concentration was expressed as millimoles of hydroxylysylpyridinoline per mole of collagen, assuming the molecular weight of collagen is 300 000.

### Immunoblotting

Muscles were lysed in ice-cold buffer containing 8.3 m urea, 2 m thiourea, 2% 3-[(3-cholamidopropyl)dimethylammonio]-1-propanesulfonate (CHAPS), 1% dithiothreitol (DTT) and were clarified at 10 000 *g* for 30 min. Aliquots (20 μg protein) were resolved by SDS-PAGE (12%), electrotransferred to Hybond-P PVDF membranes (Dutscher, Brumath, France) and probed with a mouse monoclonal anti-endorepellin (A74; Abcam) diluted (1:200) with 5% milk in 50 mm Tris–HCl (pH 8.0), 150 mm NaCl, and 0.1% Tween 20. Primary antibody was resolved using a peroxidase-conjugated secondary antibody (1:5000) and ECL Plus system (GE Healthcare, Velizy-Villacoublay, France). Signals recorded were quantified using quantity one software (Bio-Rad, Marnes La Coquette, France) and normalized against the amount of proteins (determined following Ponceau Red staining) to correct for uneven loading.

### Statistical analysis

All data are expressed as means ± SEM. Statistical analyses of each dependent variable were carried out using one-way ANOVA. When the data did not satisfy the normality criterion, the Kruskal–Wallis method was used. Multiple comparisons of the honestly significant differences were assessed by Fisher’s test. The *P* value < 0.05 was used as the basis for the conclusion of significant difference.
